# An Audit on the Safety and Efficiency of Irrigation Fluid Disposal in Urology: Time to Implement Closed Waste Management Systems?

**DOI:** 10.7759/cureus.73939

**Published:** 2024-11-18

**Authors:** Dhruv Patel, Kayzia Bertman, Jonathan Ord

**Affiliations:** 1 Urology, Cheltenham General Hospital, Cheltenham, GBR; 2 Surgery, Cheltenham General Hospital, Cheltenham, GBR

**Keywords:** closed irrigation, endo urology, surgical nursing, theatre efficiency, waste management

## Abstract

Efficient and safe disposal of irrigation fluid is critical in urological procedures due to high fluid volumes and exposure risks. This audit examines disposal practices using bucket and suction canister systems in a high-volume urology department in the UK and compares the findings with the potential implementation of an automated irrigation waste management system such as the Neptune® 3 (Stryker Corporation, Kalamazoo, Michigan).

A retrospective audit of 224 procedures at Cheltenham General Hospital evaluated fluid disposal impacts on staff safety, operating room efficiency, and cost-effectiveness. We found that procedures using high volumes of irrigants, such as transurethral resection of the prostate (TURP) and percutaneous nephrolithotomy (PCNL), are associated with increased risks, including fluid spills, staff exposure to biohazards, and significant use of single-use plastics, resulting in elevated operational costs and frequent procedural interruptions.

The Neptune® system proposes significant advantages, such as reducing fluid handling time, staff exposure, and plastic waste. Cost analysis suggests that initial investments in Neptune® would be offset by reduced consumable usage and increased efficiency. These findings support the implementation of closed fluid management systems to improve safety, efficiency, and environmental sustainability in endoscopic urology.

## Introduction

In high-volume urology departments, the management of irrigation fluids is a crucial aspect of endoscopic procedures, including transurethral resection of the prostate (TURP), transurethral resection of bladder tumour (TURBT), and ureteroscopy [1]. Effective irrigation fluid handling is vital for maintaining clear surgical views and ensuring procedural efficiency. Traditionally, irrigation fluid collection has been managed using buckets and suction canisters, which, while functional, present a number of safety hazards and operational inefficiencies [2].

Bucket and suction canister systems are commonly employed in urology theatres to collect irrigation fluids during procedures. Buckets are typically used as reservoirs to capture fluids drained through gravity or low-pressure suction, while suction canisters are employed to store larger volumes of irrigation fluid extracted during surgery [3]. Despite their prevalence, these systems pose significant risks to both staff and patient safety, as well as being inherently inefficient in high-volume settings [2].

One major hazard associated with buckets and canisters is the potential for fluid spillage during manual handling, which can lead to contamination, infection risk, and slips or falls in the operating room [4]. Additionally, the disposal of large volumes of fluid manually increases the likelihood of staff exposure to blood and bodily fluids, creating infection control concerns. Furthermore, frequent manual changes of suction canisters during long procedures can disrupt surgical workflow, contributing to inefficiencies in theatre time management [5].

From an operational perspective, the reliance on buckets and canisters is labour-intensive and prone to human error [4]. The need to manually empty or switch canisters often leads to procedure delays, impacting overall productivity in high-demand hospitals. Moreover, staff are required to don correct personal protective equipment, including a visor, mask, apron and gloves, which contributes to the increasing use of single-use plastics [6].

Given these issues, it is essential to explore alternative, more efficient methods of irrigation fluid management that can reduce the risks and inefficiencies associated with the current system [2]. This audit aims to evaluate the usage of bucket and canister systems in our district general hospital and to identify potential areas for improvement to optimise safety and efficiency in urology procedures.

Neptune® 3

The Neptune® (Stryker Corporation, Kalamazoo, Michigan) is a specialised fluid waste management system designed by Stryker to improve safety and efficiency during surgical procedures that involve large volumes of irrigation fluid [7]. It offers a closed, automated system that collects and disposes of surgical waste, including fluids and aerosols, without manual handling. The Neptune® features an easy-to-use electromagnetic attachment system that simplifies the connection to its docking station. After a procedure, it can be effortlessly wheeled over to the docking station, where the electromagnetic attachment ensures a secure connection without manual adjustments. This system enables the automatic transfer and disposal of waste fluids [7].

## Materials and methods

This retrospective snapshot audit evaluated endoscopic urological procedures in Cheltenham General Hospital, UK, from March 1 to 31, 2024. We included all rigid cystoscopies, TURBTs, TURPs and percutaneous nephrolithotomies (PCNL). We excluded Aquablation and Holmium laser enucleation of prostate procedures as the yearly numbers of these procedures were negligible.

We collected data regarding the usage of suction canisters, incontinence pads, suction tubing, suction connectors, nitryl gloves, plastic aprons and time taken to empty suction canisters. We were then able to analyse the costs associated with these products per case.

Data was entered into Excel (Microsoft, Redmond, Washington) spreadsheets, and any patient-identifiable data was anonymised. Basic statistical analyses and generation of graphs were carried out via Microsoft Excel.

## Results

Two hundred twenty-four endoscopic urology cases were carried out in our hospital in March 2024. Three PCNLs, 48 TURPs, 55 TURBTs, 105 Rigid cystoscopies and 13 ureteroscopies.

**Figure 1:**
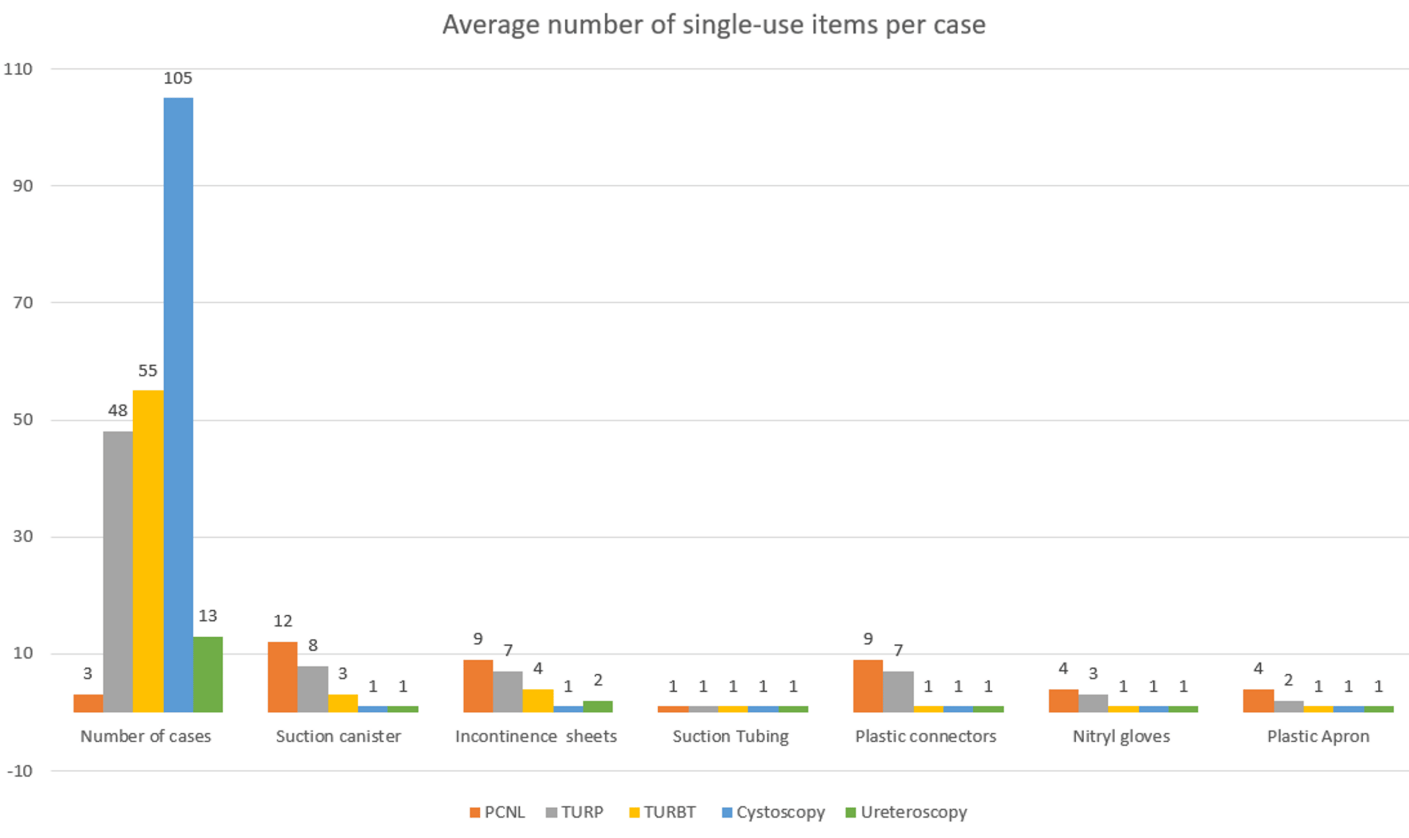
Graph of single-use item usage per case Image credit: author Kayzia Bertman PCNL - percutaneous nephrolithotomy; TURBT - transurethral resection of bladder tumour; TURP - transurethral resection of prostate

Our results demonstrate that TURPs and PCNLs use an average of eight and 12 suction canisters, respectively, per case, thus using much more irrigant than all other endoscopic urology procedures, which use one to three canisters per case. These two procedures were also associated with greater use of all single-use equipment, including incontinence sheets (for cleaning spills), suction tubing, plastic connectors, nitryl gloves and plastic aprons over the course of one month (Figure 1).

**Figure 2:**
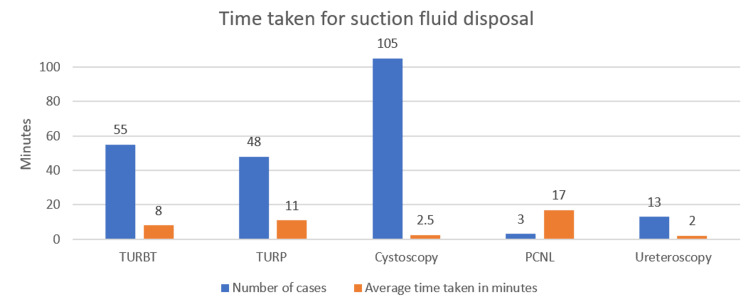
Average time per case for fluid disposal Image credit: author Kayzia Bertman PCNL - percutaneous nephrolithotomy; TURBT - transurethral resection of bladder tumour; TURP - transurethral resection of prostate

The results also showed that more time was spent changing and disposing of suction canisters during PCNLs and TURPs compared to the other endoscopic procedures (Figure 2).

Based on the data, the following cost analyses were undertaken to determine the overall financial impact associated with fluid disposal during such procedures (Table 1).

**Table 1:** Breakdown of average costs of single use items per case PCNL - percutaneous nephrolithotomy; TURBT - transurethral resection of bladder tumour; TURP - transurethral resection of prostate

Operation	Suction canister		Incontinence sheet		Suction tubing		Plastic connectors		Nitryl gloves		Plasticapron		Suction canister waste cost	Granules (per suction canister)	Total cost per case
	Total	Cost	Total	Cost	Total	Cost	Total	Cost	Total	Cost	Total	Cost			
PCNL	12	£14.28	9	£1.44	1	£0.65	9	£4.86	4	£0.08	4	£0.24	£8.16	£1.56	£31.27
TURP	8	£9.52	7	£1.12	1	£0.65	7	£3.78	3	£0.06	2	£0.12	£5.44	£1.04	£21.73
TURBT	3	£3.57	4	£0.64	1	£0.65	1	£0.54	1	£0.02	1	£0.06	£2.04	£0.39	£7.91
Rigid cystoscopy	1	£1.19	1	£0.16	1	£0.65	1	£0.54	1	£0.02	1	£0.06	£0.68	£0.19	£3.49
Ureteroscopy	1	£1.19	2	£0.32	1	£0.65	1	£0.54	1	£0.02	1	£0.06	£0.68	£0.19	£3.65

## Discussion

This audit has highlighted significant inefficiencies and safety risks associated with the current use of suction canisters and buckets during endoscopic procedures. This section discusses the findings from the audit, compares the current system with the proposed Neptune® system, and presents a strong case for its adoption based on both safety and efficiency improvements.

Safety concerns

The manual handling of suction canisters and buckets in procedures such as TURP and PCNL poses serious safety risks to staff [2]. The audit results show that a typical TURP procedure uses a median of eight canisters, with each canister containing approximately 16 litres of fluid, equating to at least 16 kg of fluid per case. This volume presents a high risk of injury, particularly given the frequent need to manually lift, carry, and dispose of the canisters [8]. Fluid spillage during the transfer of canisters or buckets also creates a slip hazard, increasing the risk of falls, contamination, and subsequent infection in the operating theatre ​[9].

The Neptune®, in contrast, eliminates much of this risk. It allows for automatic disposal of fluid without manual handling, significantly reducing the likelihood of spills and minimising staff exposure to potentially infectious waste [7]​. The current system not only exposes staff to blood, urine, and saline but also increases the risk of damaging sensitive equipment, such as camera cables, which can come into contact with spilled fluid on the floor [2].

Efficiency and theatre workflow

From an operational efficiency perspective, the data demonstrates that procedures such as TURP and PCNL require considerable time to manage irrigation fluids, with an average of 11 minutes per TURP case for a staff member to empty and dispose of the canisters. This time adds up across multiple procedures, particularly in high-volume settings, leading to substantial inefficiencies in theatre turnaround times [10].

The Neptune® on the other hand, reduces this time to a single minute per case. The device can simply be wheeled to a docking station, where it automatically empties the waste fluid, allowing staff to swiftly move to their next task [2,7]. Over time, this reduction in fluid disposal time could increase theatre efficiency, shorten procedure times, and lead to smoother workflow.

Cost Implications

In terms of cost, the audit of current suction canister use shows that TURP and PCNL cases incur substantial expenses related to single-use equipment. For example, a single PCNL case uses an average of 12 suction canisters, resulting in a total cost of £31.27 per case; a TURP procedure costs approximately £21.73 per case. These figures include the cost of suction canisters, incontinence sheets, suction tubing, connectors, nitryl gloves, and aprons.

By adopting the Neptune®, these costs can be significantly reduced. Although the initial investment in the Neptune® system is higher, the reduction in single-use plastics and associated waste management costs will likely result in long-term savings [2]. While the Neptune® system's manifold connectors cost £20 per case, this is comparable to the average cost of suction canisters per TURP or PCNL case (£21.27 to £31.27). The added benefit of reduced labour costs and improved efficiency further strengthens the financial argument for adopting Neptune®, especially for use in cases that require a high volume of irrigant.

Environmental and regulatory compliance

Additionally, the use of Neptune® aligns with broader environmental goals. The Royal College of Surgeons' Green Theatre Checklist advocates for a reduction in plastic waste [11]. By extrapolating our data, this audit reveals that nearly 5000 single-use, two-litre canisters are discarded annually. By switching to Neptune®, this figure could be reduced to just 600 smaller, single-use plastic connectors per year​. This significant reduction in plastic waste not only benefits the environment but also demonstrates a shift in attitudes in the surgical world to more sustainable healthcare practices [12].

Limitations

This study has several limitations that may affect the generalisability of its findings. Firstly, the data was collected over a single month, which may not fully capture variations in procedural volume, equipment usage, or cost fluctuations that occur over a longer period of time. Additionally, this audit was conducted within a single centre, and differences in surgical practices, staffing, or resource availability at other institutions may yield different outcomes. Finally, due to the observational nature of this audit, causal conclusions cannot be made regarding the Neptune system's impact on outcomes; a prospective study would be needed to more rigorously evaluate its efficacy.

## Conclusions

In conclusion, the data from our audit makes a compelling case for transitioning to the Neptune® system. The current use of buckets and suction canisters in urology theatres presents significant risks to staff safety, reduces theatre efficiency, and generates unnecessary waste. The Neptune® offers a potential solution that addresses all of these concerns, providing safer, faster, and more cost-effective fluid management. Adoption of this system would ensure that our urology department continues to operate at the highest standards of safety, efficiency, and environmental responsibility.
